# Isolation and Characterization of Exosomes from Cancer Cells Using Antibody-Functionalized Paddle Screw-Type Devices and Detection of Exosomal miRNA Using Piezoelectric Biosensor

**DOI:** 10.3390/s24165399

**Published:** 2024-08-21

**Authors:** Su Bin Han, Soo Suk Lee

**Affiliations:** Department of Pharmaceutical Engineering, Soonchunhyang University, Asan 31538, Republic of Korea; 1gkstkfkd@naver.com

**Keywords:** exosome, microRNA, MCF-7 cell line, surface acoustic wave (SAW) biosensor, immunoaffinity separation, paddle screw

## Abstract

Exosomes are small extracellular vesicles produced by almost all cell types in the human body, and exosomal microRNAs (miRNAs) are small non-coding RNA molecules that are known to serve as important biomarkers for diseases such as cancer. Given that the upregulation of miR-106b is closely associated with several types of malignancies, the sensitive and accurate detection of miR-106b is important but difficult. In this study, a surface acoustic wave (SAW) biosensor was developed to detect miR-106b isolated from cancer cells based on immunoaffinity separation technique using our unique paddle screw device. Our novel SAW biosensor could detect a miR-106b concentration as low as 0.0034 pM in a linear range from 0.1 pM to 1.0 μM with a correlation coefficient of 0.997. Additionally, we were able to successfully detect miR-106b in total RNA extracted from the exosomes isolated from the MCF-7 cancer cell line, a model system for human breast cancer, with performance comparable to commercial RT-qPCR methods. Therefore, the exosome isolation by the paddle screw method and the miRNA detection using the SAW biosensor has the potential to be used in basic biological research and clinical diagnosis as an alternative to RT-qPCR.

## 1. Introduction

Exosomes are small (average diameter 30–150 nm) extracellular vesicles produced by almost all cell types in the human body, and they serve to promote intercellular communication and immunomodulatory functions. In most cases, cancer cells contain higher concentrations of exosomes than healthy cells [1,2]. Current research on the development of exosome isolation technology have been reported using size exclusion chromatography, ultracentrifugation, density gradient centrifugation, and immunoaffinity exosome capture [3,4,5,6,7,8]. The ultracentrifugation method, the most common of these methods and considered the gold standard for exosome isolation, is simple but requires large amounts of samples and expensive, high-end equipment, such as a high-speed centrifuge. Therefore, it is important to obtain more efficient exosome isolation technologies that exhibit high sensitivity and high throughput. In that respect, immunoaffinity exosome capture is evaluated as an ideal method for isolating pure exosomes based on the principle of immunoaffinity interaction between exosome surface proteins (antigens) and target antibodies. It can isolate specific types of exosomes according to their surface markers [9]. However, the immunoaffinity exosome capture method often suffer from difficult exosome release [10]. Exosome contents include unique mRNAs, microRNAs (miRNAs), DNA fragments, lipids, and proteins, which genetically specify the cell of origin [3]. The most notable fact is that miRNAs exist in exosomes and, moreover, exosomal miRNAs are considered as biomarkers for many serious diseases [11]. Exosomes from diseased people contain miRNAs not found in healthy individuals [12].

MicroRNAs are small (21–25 nucleotides in length) endogenous non-coding naturally occurring single-stranded RNA molecules. Previous studies have reported that the abnormal expression of specific miRNAs is closely associated with serious human diseases, such as cancer and cardiovascular diseases. Therefore, miRNAs can be used as biomarkers for the early detection of several diseases and are also valuable therapeutic targets for the treatment of these diseases [13]. The overexpression of miRNA-106b (miR-106b) has been noted in several tumor types. Abnormal miR-106b expression is associated with breast cancer, lung cancer, gastric cancer, prostate cancer, colorectal cancer, hepatocellular carcinoma, and esophageal squamous cell carcinoma [14,15,16]. However, detecting miRNAs including miR-106b is technically difficult because they are small in size, low in abundance, and easily degradable. Therefore, accurate and sensitive miR-106b detection is important not only for understanding the biological roles of miRNAs, but also for the early and rigorous diagnosis of various human cancers.

Among various approaches to detect miRNAs, RT-qPCR is preferred as the most convenient and practical method to detect miRNAs with high sensitivity and accuracy, and several technologies have been commercialized [17,18]. Recently, miRNA detection using various types of biosensor technology (fluorescence, Electrochemistry, Organic Electrochemical Transistor (OECT), Quartz Crystal Microbalance (QCM), Surface Plasmon Resonance (SPR), Surface-enhanced Raman Spectroscopy (SERS), etc.) has been reported [19,20,21,22,23,24,25,26,27,28,29,30]. Although some DNA analysis studies have been conducted using surface acoustic wave (SAW) biosensors [31], only our previous study has reported studies on miRNA detection using SAW biosensors [32]. In this paper, three types of miRNAs (miR-21, miR-155, and miR-106b), whose upregulation is known to be highly associated with cancer, were simultaneously detected using the SAW biosensor array mounted with four individual sensors (three working sensors and a reference sensor). In particular, it is noteworthy that the reproducibility of CVs in the detection of each miRNA was improved due to normalization through the internal reference sensor.

A SAW biosensor is a piezoelectric biosensor based on proprietary surface acoustic waves to measure biomolecular interactions on the sensor surface in real-time [33,34,35,36]. Specifically, this sensor measures changes in mass as a result of biomolecular interactions that occur on the sensor surface. The SAW sensor consists of a piezoelectric substrate as the base material and an input- and output-interdigitized transducer (IDT) on both sides of the substrate. When used as a biosensor, a waveguide layer is deposited on an IDT-patterned substrate for operation in aqueous conditions. Silicon dioxide (SiO_2_) has been commonly used as a waveguide layer due to its high chemical and mechanical resistance, low acoustic loss, and the ease of the functionalization of a variety of biomaterials by the self-assembled monolayer techniques of silane compounds. The attractive advantages of the SAW biosensor, which measures the mass changes in real-time in detecting biological substances such as miRNAs, include label-free detection and simplicity of use. Additionally, signal amplification through mass increase using the gold staining of AuNPs can dramatically increase the sensitivity of detection.

This study demonstrates a SAW biosensor for the detection of miR-106b isolated from cancer cells based on immunoaffinity separation technique using our unique paddle screw-type devices. To improve detection reproducibility, an internal reference sensor was introduced in our SAW biosensor. This enables differential or normalized data acquisition from the working sensor signal and reference sensor signal, which can compensate for unexpected signal drift or noise and discriminate against non-specific binding. Normalization (working sensor response divided by reference sensor response) can also be used to suppress disturbances known to similarly influence signals of both the working and reference sensors.

## 2. Materials and Methods

### 2.1. Reagent and Materials

HPLC-purified synthetic miRNAs (miR-21, miR-124, miR-106b, and miR-155), 5′-amine modified oligonucleotide probes, and 3′-thiol modified detecting oligonucleotide probes were obtained from Bioneer (Daejeon, Republic of Korea). Their sequences are listed in Table 1. Gold nanoparticles (AuNPS, 30 nm, stabilized suspension in citrate buffer), analytical grade gold(III) chloride, sodium citrate, and hydroxylamine hydrochloride for gold staining reaction were obtained from Sigma-Aldrich Chemical Co. (St. Louis, MO, USA). (3-Glycidoxypropyl)triethoxysilane (3-GPTES), 3-amino-1-propanol, mercury(II) chloride, and dithiothreitol (DTT) were purchased from Sigma-Aldrich. Human breast total RNA (AM6952) was obtained from Thermo Fisher Scientific (Waltham, MA, USA). MCF-7 human breast cancer cell line (ATCC^®^ HTB-22™) was obtained from the American Type Culture Collection (ATCC, Rockville, MD, USA). An RNeasy Mini Kit for extracting total RNA was brought from Qiagen (Valencia, CA, USA). An HB miR Multi Assay kit™ for RT-qPCR assay to detect miRNAs was purchased from HeimBiotek, Inc. (Pangyo, Republic of Korea). The conjugation of thiol-modified detecting probes to AuNPs (30 nm) was performed using the published procedures with slight modification [37].

### 2.2. Design and Fabrication of the SAW Sensor

**Figure 1:**
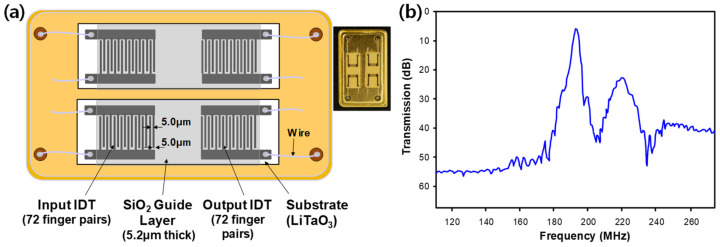
(**a**) Top view of the dual-type SAW sensor and a picture of the packaged SAW sensor. (**b**) Measured S_21_ of the SAW sensor fabricated on the LiTaO_3_ substrate in air at 25 °C.

Two pairs of IDT electrodes forming a 2-port SAW delay line were patterned onto a 36°YX-LiTaO_3_ piezoelectric substrate (Yamaju Ceramics Co., Ltd., Anada-Cho Seto City, Japan), which has been widely used due to its large electromechanical coupling factor (K^2^) with low propagation and insertion loss [38]. Higher center frequency and low insertion loss will lead to a higher sensitivity of the SAW sensor. Aluminum input- and output-IDT electrodes consisted of 72 finger electrode pairs with a width of 5.0 μm and a center-to-center separation of 10.0 μm. The spacing between delay lines was 100 λ. The area of the SAW sensor was 3.0 mm × 9.0 mm and the aperture of the IDT electrodes was 1.6 mm. This configuration allowed transmission (S_21_) and reflection (S_11_) measurements. The SAW sensor response was measured in terms of the insertion loss of the S_21_ transmission parameter. In order to confine the acoustic energy near the surface and protect the IDT electrodes from the test buffer, a 5.2 μm thick SiO_2_ guide layer determined through simulation was deposited on the sensor surface [33]. The fabricated SAW sensor could operate at a canter frequency of approximately 200 MHz. After dicing the fabricated wafer, SAW sensors were mounted in individual packages to form a dual-type SAW sensor, followed by aluminum wire bonding to establish an electric connection. Figure 1a presents a detailed configuration of the dual-type SAW sensor. Figure 1b shows the frequency response of the SAW sensor. A resonant frequency of 196 MHz was obtained, which was close to the theoretical value of 200 MHz. Then, the 3-GPTES-modified SAW sensor chip was prepared by the general silanization protocol described in Supplementary Materials (Figure S1).

### 2.3. Sensing and Fluidic Blocks of the SAW Sensor

In the sensing block, the SAW sensor was in contact with a custom-made oscillator circuitry. The mass loading effects on the SAW sensor were monitored by tracking the frequency of SAWs at the center frequency of 200 MHz with an HP8753ES network analyzer (Hewlett-Packard Company, Palo Alto, CA, USA). The instrument was optimized to measure the S_21_ frequency response of the SAW sensor. A temperature controller was installed below the chamber to keep the temperature at 25 °C. The sensing block, where the SAW sensor was to be mounted, possessed a two-part plastic fixture. The bottom piece supported the SAW sensor in a recessed cavity. It was in contact with the oscillator circuitry. The upper piece contained recessed areas for reaction chambers and silicone gaskets to prevent liquid leakage. The sample and the buffer solution that flew to the reaction chamber were actuated with a peristaltic pump (ISM597; ISMATEC, Glattbrugg, Switzerland). Through careful optimization, the flow rate was kept at 1.0 mL/min and the volume of each reaction chamber was 30 μL. The detailed configuration of the fluidic cell is provided in the Supplementary Materials (Figure S2).

### 2.4. Detection of Synthetic miR-106b Using SAW Biosensor

For miR-106b detection using the SAW biosensor, a hairpin loop structure was first formed through the addition of Hg^2+^ ion (HgCl_2_, 10 μM) on the working sensing area. After 5 min, various concentrations (0.1 pM to 1.0 μM) of synthetic miR-106b in 1X SSC buffer (pH 7.0) were added to the sensor surface and allowed to stand at 25 °C for 10 min to induce the formation of a hybrid duplex between the miR-106b and the hairpin loop-oligonucleotide probe on the surface. A DNA detection probe conjugated with AuNPs was then introduced to the partially hybridized sensor surface and allowed to stand at 25 °C for 10 min to form a sandwich hybridization. After washing with 1X SSC buffer solution for 1 min, a gold staining solution consisting of gold(III) chloride (10 mM, 50 μL) and hydroxylamine hydrochloride (20 mM, 50 μL) was added to the sensor surface and incubated at 25 °C for 2 min. The staining reaction was then stopped by washing the sensing surface with 1X SSC buffer. Identical experiments were also performed using the control miRNAs (miR-21, m-R-124, and miR-155). Meanwhile, in the reference sensing area, the same process as in the working sensing area was applied using a reference sensing probe (poly G_10_) conjugated with AuNPs.

### 2.5. Preparation of Antibody-Conjugated Paddle Screw-Type Devices for Exosome Isolation

**Figure 2:**
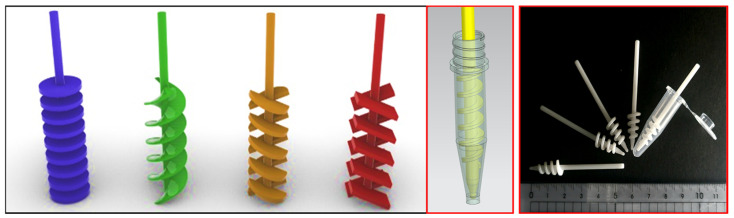
Various shapes of 3D paddle structures for the isolation of exosomes. The paddle screw shown in the red box was selected considering that it was convenient to manufacture with 3D printing and that it matched well with a 1.5 mL microtube.

Paddle screws made of acrylonitrile butadiene styrene (ABS) were manufactured using a 3-D printing method. Among the various shapes of 3D paddle structures, the paddle screw shown in the red box in Figure 2 was selected because it was convenient to manufacture by 3-D printing. In addition, it matched well with a 1.5 mL tube. They were washed by sonication in ethanol and double-distilled water (each 3 times × 5 min at room temperature), dried under nitrogen, and stored in a desiccator until use. These cleaned paddle screws were activated in a UV-ozone cleaner (144AX-220; Jelight Company, Inc., Irvine, CA, USA) for 10 min. The activated paddle screws were placed in a freshly prepared 3% (vol./vol.) 3-GPTES in ethanol for 1 h, then rinsed with ethanol for 2 min and dried under nitrogen. The silanized paddle screws were kept in an oven at 110 °C for 1 h, then rinsed with ethanol for 2 min and dried under nitrogen. For the immobilization of antibodies on the surface of paddle screws, 3-GPTES modified paddle screws were soaked in a mixed solution of anti-CD9 and anti-CD81 antibodies (each 100 μg/mL in pH 7.4 phosphate-buffered saline (PBS)). These paddle crews were incubated at 4 °C overnight. After incubation, the paddle crews were rinsed and rotated at 500 rpm with PBS and deionized water to remove physically absorbed antibodies. Sequentially, the empty sites where antibodies were not immobilized within the sensing area were blocked with 3% BSA in pH 7.4 PBS solution to prevent the non-specific binding of proteins in the subsequent steps.

### 2.6. Isolation and Characterization of Cancer Cell-Derived Exosomes

**Figure 3:**
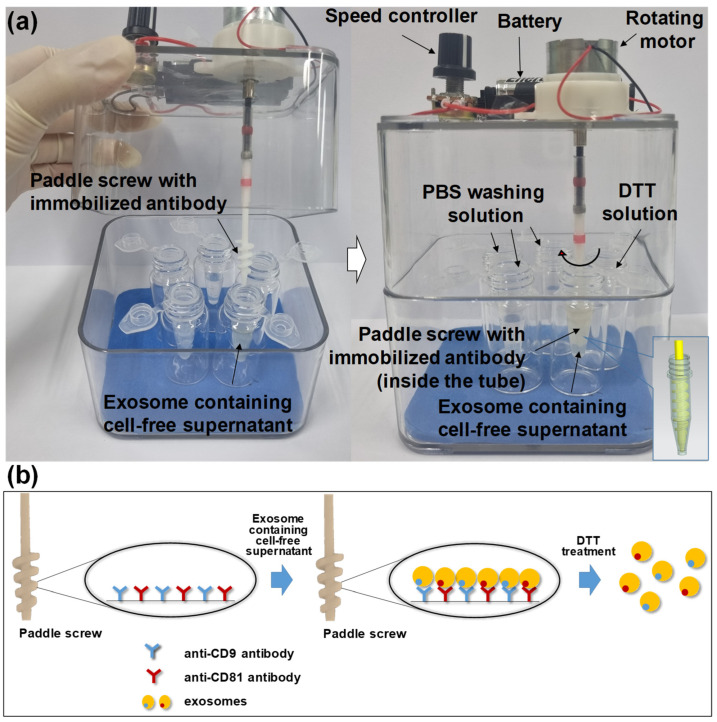
(**a**) A custom-made paddle screw-rotating device. The tubes located at the bottom of the device consisted of one exosome containing cell-free supernatant, three PBS buffer solutions, and one DTT solution. (**b**) A graphical scheme of the principle of exosome isolation by paddle screw devices.

The isolation of exosomes from an MCF-7 human breast carcinoma cell line was performed via two different immunoaffinity capture methods. The isolation using Dyna Beads^®^-CD9 and CD81 (Invitrogen, Carlsbad, CA, USA) followed the manufacturer’s instructions. Exosome isolation using paddle screws was accomplished according to the following protocol: After the MCF-7 cells were cultured for 24 h, the harvested cell culture media were centrifuged at 2000× *g* for 10 min and then at 15,000× *g* for 15 min to remove floating cells and cellular debris, followed by syringe filtration through a 0.22 µm membrane (Millipore, Billerica, MA, USA). The cell-free supernatant was then immediately transferred to a 1.5 mL tube. After adding the antibody-modified paddle crew to the tube, the paddle screw was rotated at 200 rpm for 30 min. The paddle crew was then transferred to a new 1.5 mL tube containing PBS buffer and washed by rotating at 500 rpm for 5 min. Next, the paddle crew was transferred to a 1.5 mL tube containing a dithiothreitol (DTT) solution (50 mM) and rotated at 200 rpm for 15 min to break the disulfide bond, thereby releasing the captured exosomes from the paddle screw. All of the above processes using the paddle screws were accomplished through a custom-made rotating system as shown in Figure 3. The exosome-released solution was centrifuged at 10,000× *g* for 1 h at 4 °C. After the centrifugation, the supernatant was aspirated and discarded. The pellet at the bottom of the tube contained the exosomes. The pellet was suspended in 1.0 mL of 1X PBS buffer for downstream analysis. Nanoparticle tracking analysis (NTA) measurements were performed using a NanoSight NS300 (Malvern Pananalytical, Malvern, UK) with specific parameters according to the manufacturer’s user manual. Captures and analysis were achieved using the built-in NanoSight Software NTA3.3.301. The protein concentrations of the exosomes were measured using a bicinchoninic acid assay (BCA) kit obtained from Thermo Fisher Scientific. Western blot was carried out to detect the expression levels of the exosomal surface markers, CD9 and CD81.

### 2.7. RT-qPCR for Detecting miR-106b Using Total RNA Extracted from MCF-7 Human Breast Carcinoma Cell Line

Total RNA was extracted from the exosomes isolated from the MCF-7 human breast carcinoma cell line using an RNeasy Mini Kit (Qiagen, Maryland, MD, USA) following the manufacturer’s instructions. The total RNA samples obtained were diluted to various concentrations (0.01 ng/mL to 100 μg/mL). An RT-qPCR assay for detecting miR-106b was conducted for each concentration of the total RNA sample using an HB miR Multi Assay kit™ (HeimBiotek, Pankyo, Republic of Korea) and the protocol provided by the manufacturer as follows: Polyadenylation and reverse transcription were firstly carried out at 37 °C for 30 min. Then, the fluorescence intensity of SYBR Green was measured to detect real-time PCR products. These tasks were performed on a T100™ Thermal Cycler and Bio-Rad CFX96™. The detailed process was described in the previous study [18].

## 3. Results and Discussion

### 3.1. Sensing Principle of miR-106b Using the SAW Biosensor

**Scheme 1:**
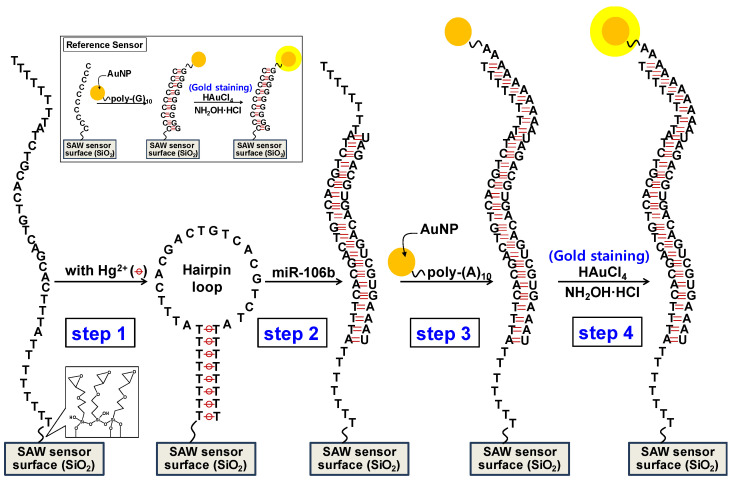
Schematic representation of sandwich hybridization and gold staining signal amplification in the working sensor for the detection of miR-106b using the SAW biosensor (the reactions occurring in the reference sensor are shown in small boxes).

Scheme 1 presents the detection of miR-106b based on sandwich hybridization between a Hg^2+^ ion-assisted hairpin loop capture probe and a target miR-106b in addition to a detection probe conjugated with AuNPs and a subsequent gold staining signal amplification on the SAW biosensor. In detail, firstly, a capture probe (5′-H_2_N-(CH_2_)_6_-TTT TTT TTA TTT CAC GAC TGT CAC GTC TAT TTT TTT T-3′) was covalently immobilized to the 3-GPTES-coated SAW sensor surface. Following the addition of the Hg^2+^ ions, consecutive thymine bases at the 3′- and 5′-ends formed a T-Hg^2+^-T pair, resulting in a stable hairpin loop structure on the sensor surface (step 1). The hairpin loop formation was almost completely achieved with the Hg^2+^ concentration at 10 μM (Figure S3). In this study, using a hairpin loop with a T-Hg^2+^-T pair as a capture probe has the following advantages: (1) it is suitable for designing oligonucleotide probes immobilized on the sensor surface for nucleic acid detection (that is, the introduction of multiple thymines at both 3′- and 5′-ends can be free from overlap with the entire miRNA sequence and can thus be applied to all the desired miRNA detection depending on sequence changes other than multiple thymines); (2) the simultaneous detection of multiple nucleic acids using an array sensor by sandwich hybridization requires only the design of a universal detecting oligonucleotide probe (poly A_10_-(CH_2_)_6_-S-AuNP in this assay); and (3) the formation and destruction of hairpin loops can be easily manipulated. Hairpin loops are easily formed by the addition of Hg^2+^ ions. Hg^2+^ ions can be removed by treatment with mercury scavengers such as thiol compounds. Thus, hairpin loops can be easily destroyed.

**Figure 4:**
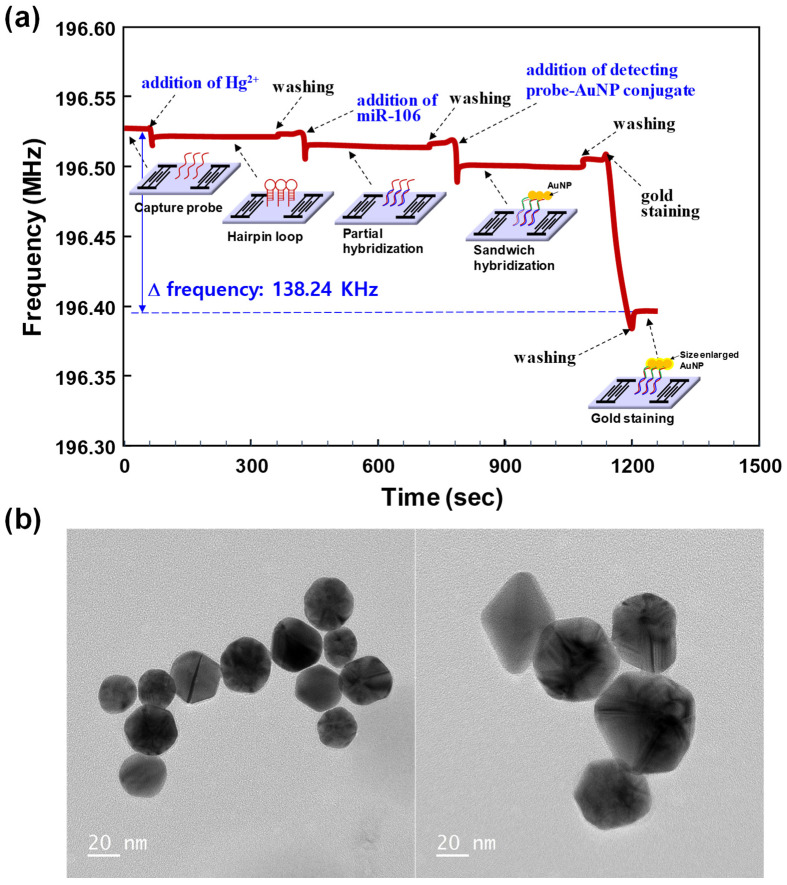
(**a**) Working sensor responses due to Hg^2+^-based hairpin loop formation, sandwich hybridization, and the subsequent gold staining reagent. A decrease in frequency indicates an increase in the effective mass of the sensor chip. A 1.0 nM concentration of the synthetic miR-106b was used in this experiment. (**b**) TEM images of AuNPs (**left**) and size-enhanced AuNPs by gold staining (**right**).

Secondly, the addition of target miR-106b can destroy the hairpin structure, resulting in partial hybridization between the capture probe and miR-106b (step 2). Sandwich hybridization then occurred between the target miR-106b and complementary two probes after introducing the oligonucleotide detecting probe with the attached AuNPs (5′-AAA AAA AAA A-(CH_2_)_6_-S-AuNP) (step 3). Finally, the size enlargement of the AuNPs by the gold staining process through reagents consisting of gold (III) chloride as an Au^3+^ source and hydroxylamine hydrochloride as a reducing agent caused a large amplification of the signal intensity (step 4). Figure 4a shows the real-time response results of the hairpin loop formation, hybridization, and signal amplification processes to detect a 1.0 nM concentration of synthetic miR-106b on the SAW biosensor surface. It was clear that gold staining was the main cause of the decrease in SAW resonance frequency. Therefore, most of the total changes in the SAW resonance frequency were generated by this signal amplification process. In this regard, we performed transmission electron microscopy (TEM) analysis to confirm the size enhancement of AuNPs by the gold staining reaction. Figure 4b shows the typical TEM images of bare AuNPs and size-enhanced AuNPs, respectively. The results of the gold staining reaction on AuNPs showed a thick deposition of metallic gold on the underlying AuNPs during the staining process. On the other hand, in the reference SAW biosensor, a capture probe [5′-NH_2_-(CH_2_)_6_-CCC CCC CCC C-3′] was immobilized and a detecting probe conjugated with AuNPs [5′-GGG GGG GGG G-(CH_2_)_6_-S-AuNP] was hybridized to the capture probe. A subsequent gold staining reaction occurred. The hybridization of poly C_10_ and poly G_10_ occurred perfectly in the reference sensor. Since the reference sensor was adjacent to the working sensors, side reactions such as non-specific adsorption that occurred in the working sensors also occurred in the reference sensor at the same level.

### 3.2. Sensitivity and Selectivity of the SAW Biosensor for miR-106b Detection

**Figure 5:**
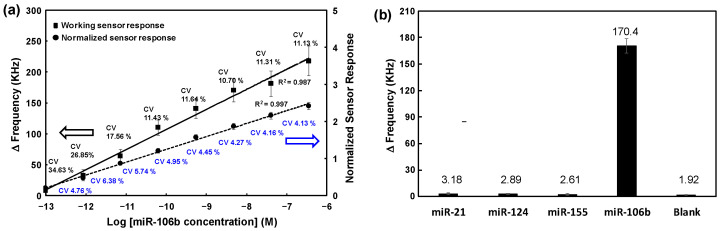
(**a**) Variations in the working sensor responses (solid line) and normalized sensor responses (dashed line) according to miR-106b concentrations. (**b**) Selectivity of the SAW biosensor toward miR-106b in comparison with the three other miRNA samples (10 μM).

A series of ten-fold serially diluted solutions at concentrations ranging from 0.1 pM to 1.0 μM of the synthetic miR-106b were prepared and the effects of concentration on SAW biosensor responses were analyzed based on the previously mentioned process and detection mechanism. As expected, as the concentration of the added miR-106b increased, the change in the resonance frequency of the SAW biosensor also increased logarithmically due to an increase in the effective mass. As the concentration of miR-106b increased, more sandwich hybridization occurred, which increased the number of AuNPs on the sensor surface. Thus, the changes in the SAW resonance frequency increased due to a mass loading effect of AuNPs and their dramatic size enlargement. The above experiments were performed in quadruplicate for each concentration of the synthetic miR-106b. The average results are shown as the solid lines in Figure 5a. Blank subtraction was performed for all the delta frequency values for each concentration of miR-106b. Here, the blank refers to the result of all the subsequent processes being performed without adding the target miR-106b to the hairpin loop formed by adding Hg^2+^ to the capture probe. The delta (Δ) frequency value of the blank sample was 2.42 ± 0.15 KHz and the limit of detection (LOD) of miR-106b was calculated to be 0.084 pM. The LOD was defined according to NORMAN guidelines based on ISO/DIS 13530 as three times the standard deviation of a blank sample (σ_blank_), a commonly accepted criterion [39].

LOD = 3 σ_blank_ (1)

In addition to sensitivity, one of the important factors in biosensor performance is target selectivity. In order to verify the selectivity of the target miRNA, we applied a target miR-106b and three control non-complementary miRNAs (miR-21, miR-124, and miR-155). The measurement results are shown in Figure 5b. The target miR-106b showed a significantly larger signal than the results for the three non-complementary targets (e.g., 53.0-fold greater signal than that of miR-155). When a mixture of the four miRNAs was applied, the change in the resonance frequency was similar to that caused by miR-106b, indicating that no significant interference occurred between them.

Meanwhile, although blank subtraction was performed to reduce deviation from different measurements, the signal deviation at each concentration of miR-106 was somewhat large as shown in the solid line in Figure 5a. Thus, we normalized the frequency changes resulting from the sandwich hybridization and a subsequent gold staining at each concentration of miR-106b to the frequency change occurring in the reference sensor. The dotted lines in Figure 5a show the normalized average results from four repeated measurements for each concentration of miR-106b. The coefficient of variation (CV) of the normalized sensor response was significantly lower than that seen from the working sensor. In detail, the CVs of the normalized sensor response ranged from 4.1% to 6.4%, while the CVs of the working sensor ranged from 10.7% to 34.6%. The high CVs of the working sensor response in this miR-106b detection assay were mainly due to the non-specific adsorption of undesired molecules and the nonlinear growth of metallic gold on AuNPs due to gold staining as well as various environmental factors known to affect piezoelectric sensors such as temperature, pressure, and viscosity. However, when normalization was performed by introducing a reference sensor adjacent to the working sensor, the CVs of the miR-106 analysis were significantly reduced, as shown in Figure 5a, and as a result, the reproducibility of the SAW biosensor was dramatically improved. This showed that normalization can filter out background noise due to environmental factors and minimize the inhomogeneity of the gold staining, which most significantly affected CVs. As an effect of increasing sensitivity as much as reducing the reproducibility of CV, the LOD of miR-106b detection using normalized signals was improved to 0.0034 pM. These results show that the LOD and linearity of the SAW biosensor for detecting miR-106b in this study are comparable to those of the recently reported miRNA biosensors (Table 2) [19,20,21,22,23,24,25,26,27,28,29].

### 3.3. Exosome Isolation from MCF-7 Cell Line and Its Characterization

**Figure 6:**
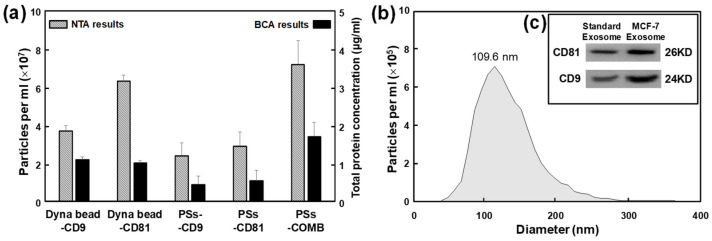
Characterization of exosomes. (**a**) NTA and BCA total protein analysis of isolated exosomes from MCF-7 cells, (**b**) particle size distribution of isolated exosomes by PSs-COMB, and (**c**) Western blotting with antibodies against CD9 and CD81 for isolated exosomes by PSs-COMB.

Here, two immunoaffinity-based approaches, commercially available magnetic beads conjugated with anti-CD9 and anti-CD81 antibodies (Dyna beads^®^-CD9 and Dyna beads^®^-CD81) and paddle screws conjugated with anti-CD9 and anti-CD81 antibody using a combination of two antibodies (PSs-CD9, PSs-CD81, and PSs-COMB), for isolating exosomes from cell-free supernatants obtained by the pre-enrichment of cultured MCF-7 cell lines were compared. The MCF-7 cell line is routinely used as a model system for human breast cancer research and the expression level of miR-106b is known to be significantly higher in MCF-7 breast cancer compared to normal breast epithelial cells. Consequently, the upregulation of miR-106b is known to promote the proliferative capacity of MCF-7 cells in vivo [40,41]. Pre-enrichment was performed by ultracentrifugation. Exosomes isolated by the two different immunoaffinity-based methods were validated by nanoparticle tracking analysis (NTA). Total protein concentration was determined using a bicinchoninic acid (BCA) assay (Figure 6a). For the MCF-7 cell line, the PSs-COMB resulted in a relatively high yield (the exosome concentration was about 7.2 × 10^7^ particles/mL) and purity of isolated exosomes. PSs-CD9 and PSs-CD81 using single CD9 or CD81 antibodies showed lower yields than those using commercially available Dyna beads^®^ for exosome isolation. However, PSs-COMB, which combined two CD9 and CD81 antibodies at a 1:1 ratio, had a higher isolation yield than using a single antibody. Its yield was similar to the commercial method. It has been demonstrated that using a combination of two types of exosome-specific antibodies rather than a single antibody is a more sensitive and specific strategy for exosome isolation and protein analysis. The results of this study showed that the paddle screw-based exosome isolation approach could offer an accessible, flexible, and efficient method, with a recovery time of less than 1 h without including a pre-enrichment step. In particular, it has an exosome isolation efficiency that is comparable to that of the commercially optimized Dyna-beads method (incubation time of 16–20 h at 4 °C). It also shows a fast analysis time. The stirring process by the active rotation of the paddle screw plays a crucial role. For example, the difference in mixing effect between two liquids resulting from simple diffusion and active stirring (200 rpm) based on computational fluid dynamics (CFD) simulations was about 13 times (Figure S4). Meanwhile, the average diameter of the isolated exosomes by the PSs-COMB method was about 109.6 nm, as detected by the NTA characterization (Figure 6b). Additionally, these isolated exosomes were validated through the quantitative analysis of CD9 and CD81 as common exosome biomarkers using Western blot. Western blot showed that CD9 and CD81 were expressed on the MCF-7-derived exosomes (Figure 6c), and these results indicated that these isolated vesicles with the paddle screws contained the exosomal proteins CD9 and CD81. Thus, they were considered as genuine exosomes.

### 3.4. Comparison of miR-106b Detection in MCF-7 Cell-Derived RNA Samples Using RT-qPCR Assay and SAW Biosensor

**Figure 7:**
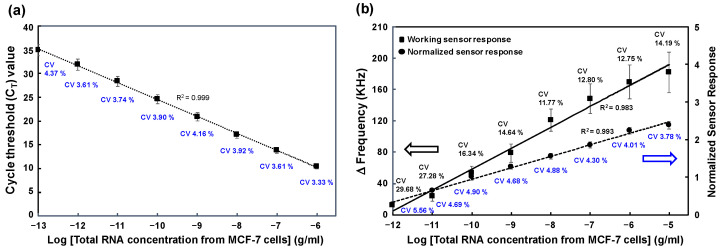
(**a**) Extrapolation of the C_T_ values obtained from the RT-qPCR assay as a function of total RNA concentration. (**b**) Variations in the working sensor responses (solid line) and normalized sensor responses (dashed line) of SAW biosensors as a function of total RNA concentration.

To confirm the performance of the SAW biosensor for analyzing the total RNA samples derived from cancer cells, we performed a miR-106b detection assay using the total RNA extracted from the exosomes isolated from the MCF-7 cell line with the PSs-COMB method. Total RNA extraction from the isolated exosomes was carried out using the RNeasy Mini Kit (Qiagen). The extracted samples were serially diluted 10-fold to obtain various concentrations (0.1 pg/mL to 10 ug/mL). Firstly, we conducted an RT-qPCR analysis using the HB miR Multi Assay kit™ for the detection of miR-106b in the total RNA extracted from the isolated exosomes. Plotting logarithmic units of the total RNA concentration versus cycle threshold (C_T_) values showed excellent linearity (correlation coefficient of 0.999) and sensitivity with an LOD of 0.028 pg/mL of total RNA (Figure 7a).

When the same samples used for the RT-qPCR were applied to the SAW biosensor, the normalized sensor responses are shown in Figure 7b. The SAW biosensor results also showed a good linearity (correlation coefficient of 0.993). The LOD was 0.064 pg/mL for total RNA extracted from isolated exosomes, which was 2.3 times higher than RT-qPCR results. RT-qPCR is an essential tool in the field of molecular biology due to its high sensitivity, specificity, and accuracy, and is currently the most practical and convenient method to detect miRNAs. It has been reported that up to less than 10 miRNA molecules can be detected. Despite the rather high LOD and error range, the SAW biosensor system can be compared to RT-qPCR in detecting miRNA due to its simple use, low equipment price, and dramatic improvement in CV values by introducing a reference sensor. Meanwhile, this result also showed good sensitivity, about 1000 times better than the experiment using the commercial human breast reference total RNA (LOD of 62 pg/mL in Figure S5). Generally, the concentration of miR-106b in the total RNA extracted from cancer cells is known to be 100 to 1000 times higher than the concentration of the total RNA extracted from healthy people [42]. Therefore, the amount of miR-106b contained in the total RNA extracted from the MCF-7 cell line was higher than the amount of miR-106b present in the same concentration of the reference human breast total RNA from healthy people. Therefore, the designed miRNA detection mechanism of the SAW biosensor worked sufficiently using the cancer cell-derived RNA samples.

## 4. Conclusions

In this study, we successfully detected miR-106b with high sensitivity and reproducibility by applying a Hg^2+^-mediated hairpin loop formation, sandwich hybridization supported by AuNP-conjugated detecting probe, and the subsequent size enlargement of AuNPs by gold staining on an SAW biosensor. Through this process, the LOD of synthetic miR-106b was 0.0034 pM, and the reproducibility of CVs were confirmed to be as low as 4.1% to 6.4% when normalization by the internal reference sensor was applied. The SAW biosensor was also highly selective for miR-106b without being significantly affected by other miRNAs. Additionally, this SAW biosensor could detect miR-106b in the total RNA extracted from the MCF-7 cancer cell line using paddle screw-type devices, showing performance comparable to that of RT-qPCR analysis. In particular, the immunoaffinity exosome capture method using our unique paddle screw device and customized rotation system quickly and efficiently isolated exosomes from the MCF-7 cancer cell line.

In brief, this methodology is easy to use, specific, and highly sensitive, showing the potential to be used as a promising alternative for the widespread detection of target miRNAs, such as miR-106b, in real samples for clinical applications. It is believed that our SAW-based miRNA biosensor and exosome isolation system are suitable for biomedical research, the early detection of malignancies, and the evaluation of the effectiveness of cancer treatment. Future research should focus on improving the performance of the paddle screw-based exosome isolation system with the ability to isolate exosomes more efficiently and the miniaturization of the paddle screw device to enable point-of-care testing. On the other hand, we plan to apply paddle screw-based devices to a wide range of practical applications such as immunoassay for disease biomarkers in human serum or plasma.

## Figures and Tables

**Table 1:** Sequences of oligonucleotides used in this study.

Oligonucleotides	Sequences
miR-21 (control)	5′-UAG CUU AUC AGA CUG AUG UUG A-3′
miR-106b (target)	5′-UAA AGU GCU GAC AGU GCA GAU-3′
miR-124 (control)	5′-CGU GUU CAC AGC GGA CCU UGA U-3′
miR-155 (control)	5′-UUA AUG CUA AUC GUG AUA GGG GUU-3′
capture probes (complementary sequence of miR-106b)	5′-H_2_N-(CH_2_)_6_-ATC TGC ACT GTC AGC ACT TTA CCC GGG CCC G-3′
reference capture probe	5′-H_2_N-(CH_2_)_6_-CCC CCC CCC C-3′
target detecting probe	5′-AAA AAA AAA A-(CH_2_)_6_-S-S-(CH_2_)_6_-OH-3′
reference detecting probe	5′-GGG GGG GGG G-(CH_2_)_6_-S-S-(CH_2_)_6_-OH-3′

**Table 2:** Comparison of LOD for miRNA detection using various detection methods.

Methods	Target	Linear Range (pM)	LOD (pM)	References
Fluorescence	miR-21	0.0005–100	0.00017	[19]
	miR-21	5–5000	1.4	[20]
	miR-155	10–400	5.0	[21]
Electrochemical	miR-21	0.001–1000	0.00002	[22]
	miR-34a	100–1,000,000	0.00094	[23]
	miR-224	1.0–1000	50	[24]
OECT	miR-21	0.001–100,000	0.01	[25]
	miR-21	5–20,000	2.0	[26]
QCM	miR-21	1000–10,000	400	[27]
SPR	miR-155	1.0–1,000,000	0.5	[28]
SERS	miR-200a	0.0001–1000	0.00026	[29]
SAW	miR-106b	0.1–1,000,000	0.0034	This work

## Data Availability

Data are contained within the article.

## Supplementary Materials

The following supporting information can be downloaded at: https://www.mdpi.com/article/10.3390/s24165399/s1, Figure S1: Optimization of the concentration of the immobilized capture probe; Figure S2: Sensing and fluidic blocks of the SAW sensor; Figure S3: Optimization of the concentration of added Hg^2+^ ions for the hairpin loop formation; Figure S4: Computational fluid dynamics (CFD) simulations of the mixing effect of two liquids; Figure S5: Detection of miR-106b in human breast total RNA.
