# Supplementary material for: Isolation and Characterization of Exosomes from Cancer Cells Using Antibody-Functionalized Paddle Screw-Type Devices and Detection of Exosomal miRNA Using Piezoelectric Biosensor
Source: Sensors (Basel). 2024 Aug 21;24(16):5399. doi: 10.3390/s24165399 (PMC11359151; doi:10.3390/s24165399)
Supplement: Supplementary file 1 [file sensors-24-05399-s001.zip › sensors-3131389-supplementary.pdf]

Supplementary Materials

Isolation and characterization of exosomes from cancer cells using antibody-functionalized paddle screw type devices and detection of exosomal miRNA using piezoelectric biosensor

Su Bin Han and Soo Suk Lee*

Department of Pharmaceutical Engineering, Soonchunhyang University, 22 Soonchunhyang-ro, Shinchang-myeon, Asan-si, Chungcheongnam-do, 31538, Republic of Korea
* Corresponding author. Tel.: +82-41-530-1394; Fax: +82-41-530-3085.
E-mail addresses: sslee0810@sch.ac.kr (S.S. Lee)

1) Optimization of the concentration of the immobilized capture probe

SiO_2 guide layered SAW sensor chips were rinsed with absolute ethanol for 2 min and washed with double distilled water. After drying under nitrogen, sensor chips were activated in a plasma chamber (Harrick Plasma, Ithaca, NY, USA) for 5 min. These activated SAW sensor chips were allowed to stand in a solution of freshly prepared 3% (vol./vol.) 3-GPTES in ethanol for 1 h, followed by rinsing with ethanol for 2 min and drying under nitrogen. These silanized sensors were oven-baked at 110°C for 1 h, followed by rinsing with ethanol for 2 min and drying in a nitrogen atmosphere. On the working sensor chip, 5'-amine-modified capture probe (5'-H_2N-$(CH_2)_6$-TTT TTT TTA TTT CAC GAC TGT CAC GTC TAT TTT TTT T-3') was attached to the surface of the 3-GPTES-modified SAW sensor chip using the following protocol: The 3-GPTES-modified SAW sensor chip was treated with 100 μM 5'-amine-modified capture probe which was adjusted for high-density immobilization dissolved in 1X SSC buffer. Excess epoxide groups of 3-GPTES were deactivated with 50 mM 6-amino-1-propanol in 1X SSC buffer for 30 min at 37 °C. The sensor chip was washed with 1X SSC buffer and double distilled water sequentially. Capture probe-modified sensor chips were then desiccated for storage at room temperature until use. In the reference sensing area, the same process as in the working sensing area was applied using a 5'-amine-modified reference capture probe (5'-H_2N-$(CH_2)_6$-poly C_{10}).

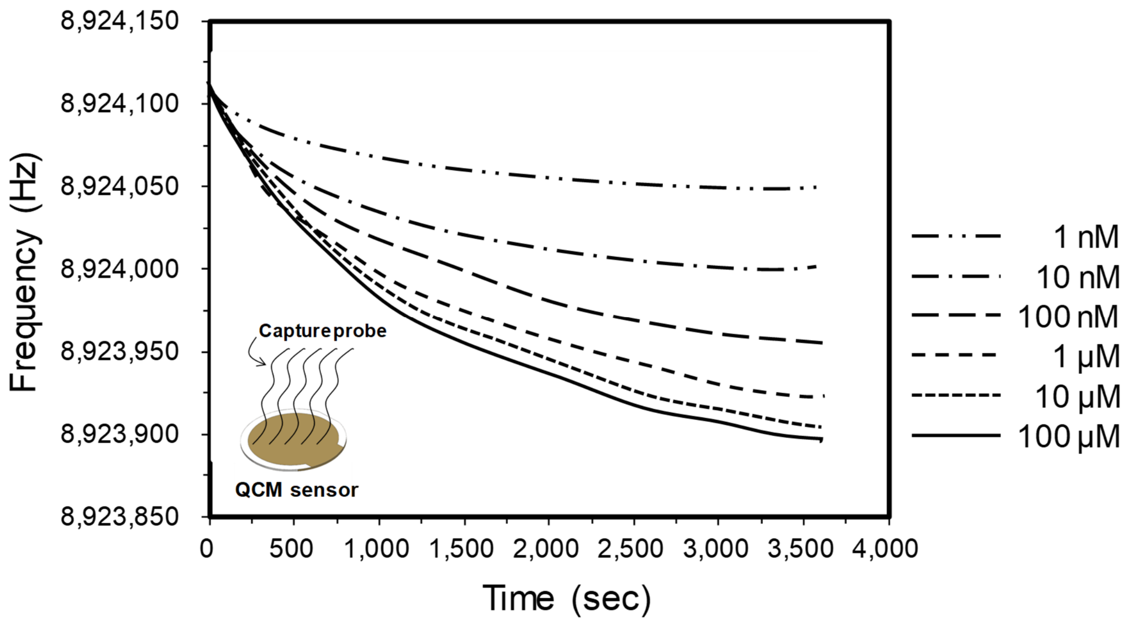

Figure S1. Concentration optimization of the immobilized capture probes tested using a 9 MHz quartz crystal microbalance (QCM) resonator. When the probe concentration reaches 100 μM, saturation begins to occur.

2) Sensing and fluidic blocks of the SAW sensor

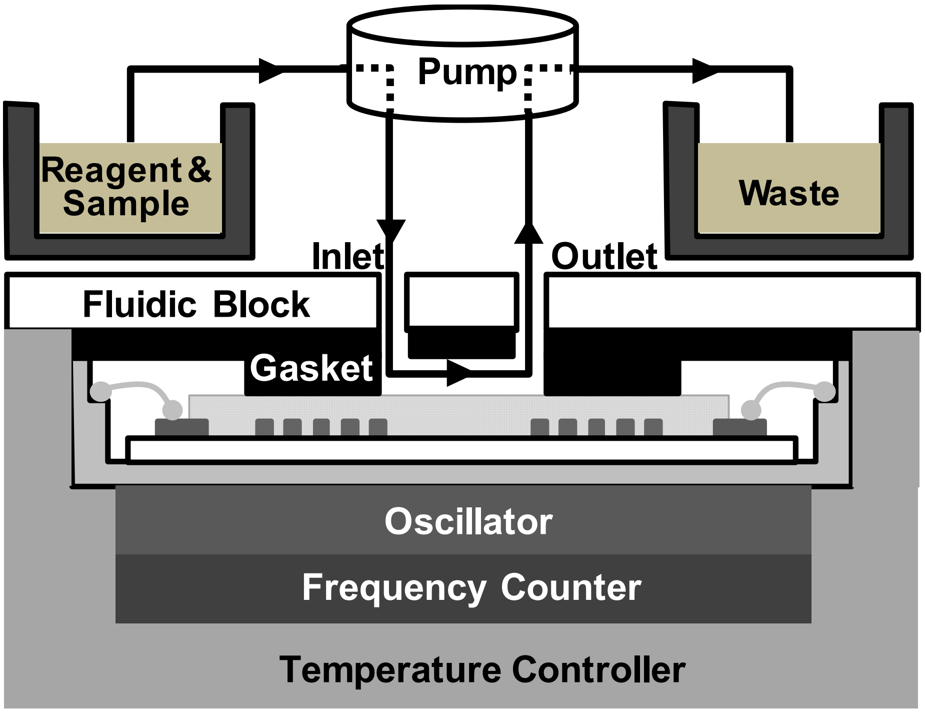

Figure S2. Configuration of the fluidic cell consisting of a peristaltic pump for fluid transfer with a custom-made fluidic block and silicon gasket for fluid control

3) Optimization of the concentration of added Hg^{2+} ions for the hairpin loop formation

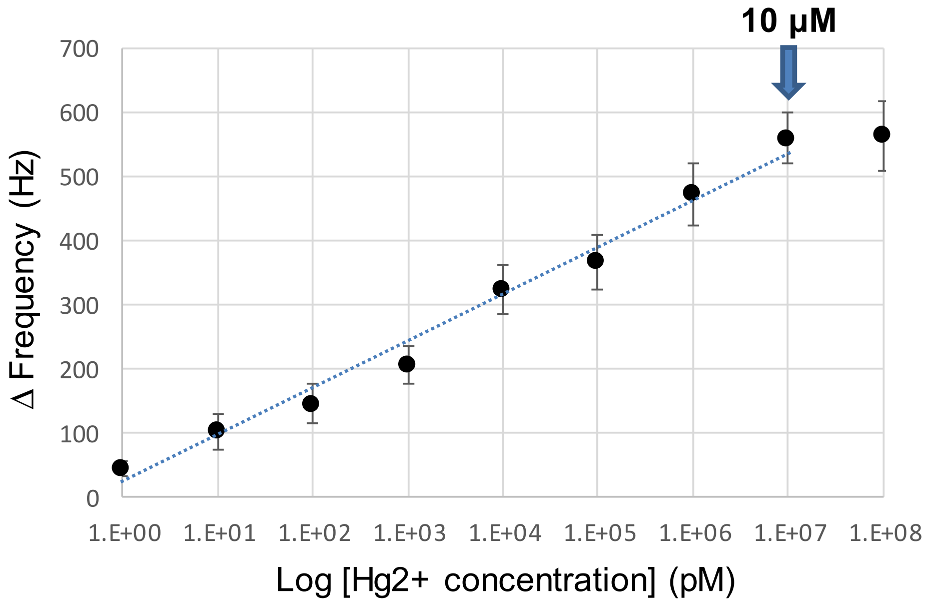

Figure S3. Concentration optimization of the Hg^{2+} ions tested using a 9 MHz quartz crystal microbalance (QCM) resonator. When the Hg^{2+} ion concentration reaches 10 μM, saturation begins to occur.

4) Computational fluid dynamics (CFD) simulations of the mixing effect of two liquids

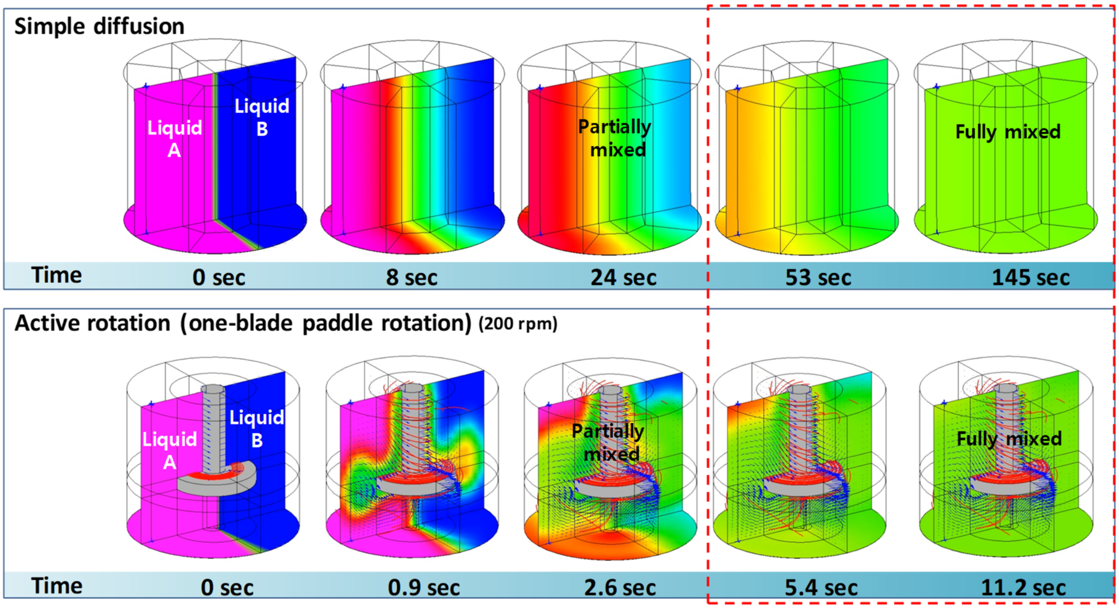

Figure S4. Computational fluid dynamics (CFD) simulations of the mixing effect of two liquids. The difference in mixing effect between the two liquids due to simple diffusion and active rotaion with a single-blade paddle (200 rpm) was approximately 13 times.

5) Detection of miR-106b in human breast total RNA

Accurate quantitative analysis of specific miRNAs in total RNA is useful for applications in both basic biology and clinical diagnosis. We analyzed effects of human breast total RNA concentrations on SAW biosensor responses for the detection of miR-106b. Experiments were also performed four times for each concentration of 10-fold serially diluted commercially purchased human breast total RNA, ranging from 1.0 ng/ml to 10 mg/ml. Average values of changes in SAW resonance frequency using various amounts of human breast total RNA for detecting miR-106b are shown in Figure S5. Results showed a good linearity (correlation coefficient of 0.985) for overall concentrations of total RNA on a logarithmic scale. When normalization using the reference sensor was performed as in the previous experiment, the minimum detectable concentration of total RNA was 62 pg/ml.

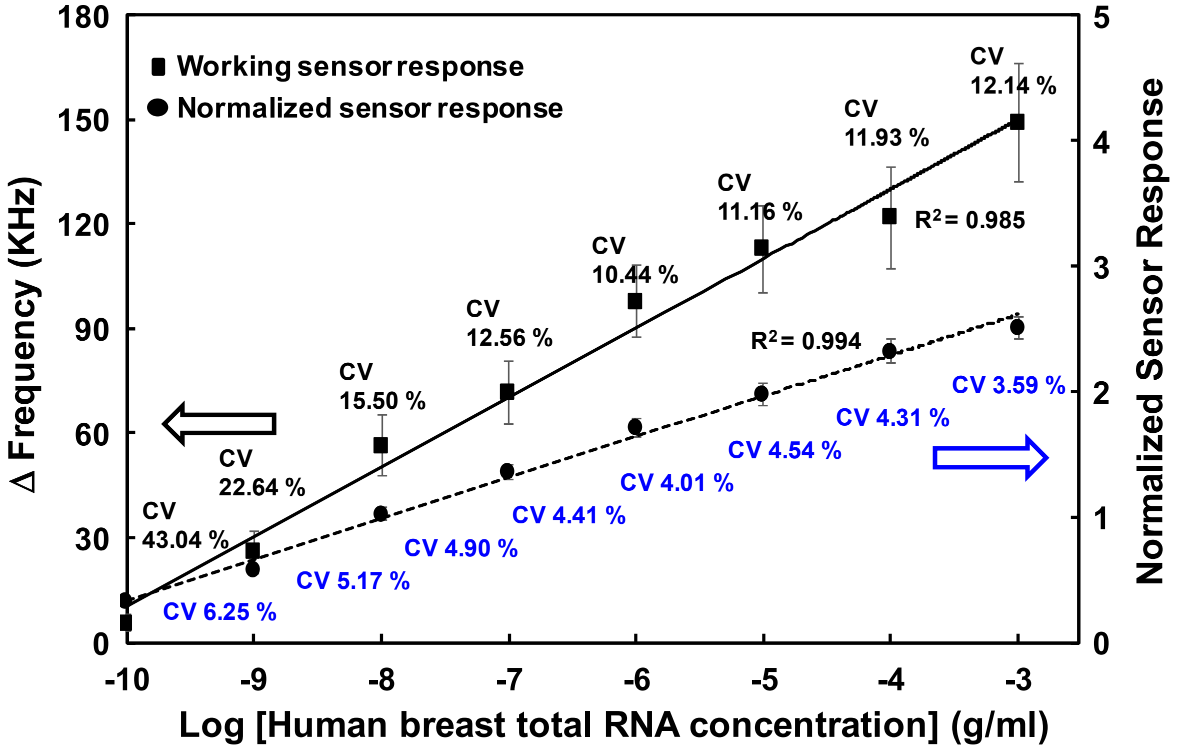

Figure S5. Variations of working sensor responses (solid line) and normalized sensor responses (dashed line) of the SAW biosensor as a function of log (total RNA extracted from the exosomes isolated from the MCF-7 cell line) concentration in the range of 1.0 pg/ml to 10 μg/ml.
